# Intestinal Stenosis Secondary to Grade IV Endometriosis: A Case Report

**DOI:** 10.7759/cureus.101965

**Published:** 2026-01-21

**Authors:** Quitzia M Rentería Fonseca, Alma D Franco Jiménez, Martha Bramasco Mendoza, Cristino Coyac Aguilar

**Affiliations:** 1 General Surgery, Hospital General "Dr. Aquiles Calles Ramírez" ISSSTE, Tepic, MEX; 2 Gynecology, Hospital General "Dr. Aquiles Calles Ramírez" ISSSTE, Tepic, MEX; 3 Coloproctology, Hospital General "Dr. Aquiles Calles Ramírez" ISSSTE, Tepic, MEX; 4 Oncology, Hospital General "Dr. Aquiles Calles Ramírez" ISSSTE, Tepic, MEX

**Keywords:** case reports, endometriosis, intestinal obstruction, intestinal resection, sigmoid colon

## Abstract

Intestinal stenosis secondary to deep endometriosis grade four is an uncommon presentation that often leads to delayed diagnosis and challenging therapeutic decisions. We report the case of a 41-year-old woman with long-standing pelvic pain, dysmenorrhea, and alternating constipation and diarrhea, with a history of surgery for endometriosis. The aim is to describe the diagnostic and therapeutic pathway and to highlight practical lessons for clinical care. Clinical assessment and blood tests revealed mild anemia. Colonoscopy showed a narrowed sigmoid colon with intact mucosa, and contrast-enhanced computed tomography demonstrated an abrupt caliber transition and focal wall thickening. Management consisted of a multidisciplinary-planned segmental intestinal resection. Histopathology confirmed endometrial glands and stroma infiltrating the muscular layer with fibrosis and no atypia. Follow-up documented improvement of obstructive symptoms and absence of immediate complications. This case contributes an integrated view of endoscopic, radiologic, and pathological findings explaining stenosis caused by extrinsic involvement of the bowel wall. The key lesson is to maintain a high index of suspicion for intestinal endometriosis in women of reproductive age with cyclic gastrointestinal symptoms and stenosis without mucosal disease, emphasizing multidisciplinary evaluation and histological confirmation to timely define surgical treatment.

## Introduction

Endometriosis is a gynecological disorder characterized by the presence of ectopic endometrial tissue outside the uterine cavity, affecting approximately 10-15% of women of reproductive age. In its deep form (classified as the American Society of Reproductive Medicine (ASRM) stage IV), lesions infiltrate the peritoneum to more than 5 mm and are usually associated with extensive retroperitoneal nodules. Clinically, it presents with intense cyclical pelvic pain, severe dysmenorrhea, dyspareunia, and infertility, symptoms that are exacerbated during menstruation. These advanced cases cause marked fibrosis and pelvic anatomical distortion, which complicates therapeutic management [1].

Bianchi et al. [2] reported that intestinal endometriosis is the most common extragenital site, affecting 3-12% of patients with endometriosis. In selected series and review articles focusing on deep infiltrating endometriosis (DIE), the prevalence of bowel involvement increases and may reach 15-20% [3]. The most commonly involved segments are the rectosigmoid colon (60-93%) and, to a lesser extent, the ileocecal junction and the appendix. This predilection explains the predominant clinical presentation in the lower gastrointestinal tract. Histopathological studies show that mucosal infiltration is rare (~10% of cases), with serous and submucosal involvement predominating, which explains the characteristics of the endoscopic findings.

Gastrointestinal symptoms can complicate the clinical picture. In addition to the classic gynecological symptoms (pelvic pain, dysmenorrhea, and dyspareunia), patients with intestinal endometriosis experience cyclical gastrointestinal symptoms. These include rectal pain during bowel movements (especially during menstruation), dyschezia, and bowel rhythm disturbances - with alternating diarrhea and constipation in approximately 14% of cases - as well as abdominal distension and tenesmus. Catamenial rectal bleeding is also common (15-20%), and symptoms of intestinal subocclusion are present in 10-12%. This combination of gynecological and gastrointestinal symptoms makes clinical recognition of the cause difficult, as it can be confused with other conditions, such as irritable bowel syndrome, inflammatory bowel disease, or colorectal neoplasia.

The diagnosis of intestinal endometriosis is often delayed due to its relatively low incidence and the overlap of symptoms with other gastrointestinal conditions. The lack of initial suspicion explains the frequent diagnostic delay and late presentation of complications. In the context of acute abdomen or intestinal subocclusion, endometriosis is rarely considered as the initial diagnosis. Bianchi et al. [2] observed that approximately 85% of patients with intestinal involvement have a history or previous clinical findings suggestive of pelvic endometriosis. This finding highlights the importance of a detailed history: severe dysmenorrhea, infertility, previous surgeries for endometriosis, or other cyclical gynecological symptoms should raise the possibility of bowel involvement.

Imaging and endoscopic studies are essential to confirm clinical suspicion. Transvaginal ultrasound (often with bowel preparation) and MRI provide details on the extent of lesions in the pelvis and bowel wall. Colonoscopy is especially useful for evaluating colonic involvement: it typically reveals extrinsic compression of the lumen with intact mucosa [4]. Colonoscopy is not recommended as a first-line diagnostic tool in suspected bowel endometriosis, given its low sensitivity, as mucosal involvement occurs in less than 10% of cases. Most lesions primarily affect the serosa and muscularis propria, resulting in normal endoscopic findings. Therefore, colonoscopy is mainly indicated in selected cases with obstructive symptoms, suspected luminal stenosis, or when differential diagnoses such as colorectal malignancy need to be excluded [5,6]. Given these findings, biopsies of the affected areas are recommended, as the differential diagnosis includes stricturing processes such as Crohn's disease or colorectal neoplasia, and definitive diagnosis relies on histopathological confirmation. Cytological analysis, including peritoneal fluid cytology, may be performed in selected cases but has limited diagnostic value in endometriosis. In emergency situations due to bowel obstruction, contrast-enhanced computed tomography can also help delineate the stricture and rule out tumoral causes.

Pathologically, advanced lesions of intestinal endometriosis can extend to the muscularis layer and, in rare cases, affect the mucosa, causing cyclic bleeding. However, the resulting fibrosis usually progresses insidiously, so complete obstruction is rare. Juárez et al. [7] reported that complete stenosis of the intestinal lumen occurs in less than 1% of cases of intestinal endometriosis. This fact indicates that the presence of significant stenosis in the context of deep endometriosis is unusual and clinically relevant. Most cases with obstructive symptoms have a prolonged history of gastrointestinal and gynecological discomfort preceding the acute finding, reinforcing the need to consider this entity in persistent differential diagnoses.

Deep endometriosis (grade IV) represents the most severe form of the disease and poses a greater surgical challenge. This stage is defined by infiltrative implants affecting the pelvic and adjacent organs with a depth of >5 mm. This infiltration causes adhesions.

## Case presentation

The patient was a woman of reproductive age with a long-standing history of endometriosis. Menarche occurred at 11 years of age, with regular menstrual cycles (28 × 4 days). She reported dysmenorrhea associated with alternating constipation and diarrhea. Cervical cytology screening had been performed annually, with all results reported as normal. Breast ultrasound examinations were unremarkable, and mammography had not yet been performed.

She reported occasional alcohol consumption until months prior to presentation and denied other toxic habits. At the time of evaluation, she was receiving antispasmodic and analgesic medications, including lysine and pargeverine, as well as mefenamic acid, which had been initiated after placement of a levonorgestrel-releasing intrauterine device. Prior to intrauterine device placement, she had used etoricoxib and dexketoprofen for pain control. She was not receiving hormonal suppression therapy at the time of presentation.

Her gynecologic history was notable for progressive pelvic pain over several years. She initially underwent exploratory surgery for suspected acute appendicitis, during which endometriotic implants were identified, and a clinical diagnosis of endometriosis was established, although without histopathological confirmation. Subsequently, pelvic ultrasonography documented a left ovarian endometrioma, which was managed conservatively for several months. Approximately one year later, surgical resection of the endometrioma with left oophorectomy was performed. Months later, a second surgical intervention was required due to persistent pelvic pain and constipation, during which additional endometriotic implants and adhesions were excised.

Following these surgical interventions, she received combined oral contraceptive therapy for approximately one year. Subsequently, treatment with a gonadotropin-releasing hormone agonist was initiated but discontinued after a few months due to significant adverse effects, including arthralgia, hot flashes, and excessive sweating. Hormonal therapy was then prescribed to restore normal menstrual cycles.

Several years later, she achieved a full-term pregnancy without complications and delivered vaginally. No infertility evaluation was performed. After pregnancy, she experienced recurrent pelvic pain, which was managed symptomatically. A hormonal intrauterine device was later placed as part of symptom management.

The patient presented hemodynamically stable, with no fever or signs of peritoneal irritation. The abdomen was mildly distended, with no palpable masses and preserved peristalsis. Gynecological examination revealed a retroverted uterus with tenderness upon cervical motion. Nodular indurations were palpated in the uterosacral ligaments and rectovaginal septum, areas that were tender to deep palpation. Similar findings have been described in cases of deep infiltrating endometriosis, including a tender mass in the upper third of the rectovaginal septum protruding into the rectum and vagina, which is comparable to the nodular indurations palpated in the rectovaginal septum in the present case, consistent with DIE. These findings suggest pelvic endometriotic infiltration with rectal involvement, consistent with the clinical picture of chronic pelvic pain and alternating constipation and diarrhea.

Diagnostic methods

Cell count: mild normocytic anemia (Hb of 11.5 g/dL, ref. range: 11.6-15.5) and hematocrit 34.6% (ref. range: 36-47%). Leukocytes (6,770/mm³) and platelets (312,000/mm³) were within normal ranges. Blood chemistry (glucose, kidney function, electrolytes) and coagulation times were normal (Table 1).

**Table 1:** Lab findings Source: Own elaboration

Lab findings		
Parameter	Result	Normal values
Hemoglobin	11.5 g/dL	11.6-15.5 g/dL
Hematocrit	34.60%	36-47%
White blood cell count	6,770/mm³	4,000-10,000/mm³
Platelets	312,000/mm³	150,000-400,000/mm³

Serum CA-125 levels were not measured, as this marker is nonspecific and was not considered essential for diagnostic or therapeutic decision-making in this case.

Imaging studies

**Figure 1:**
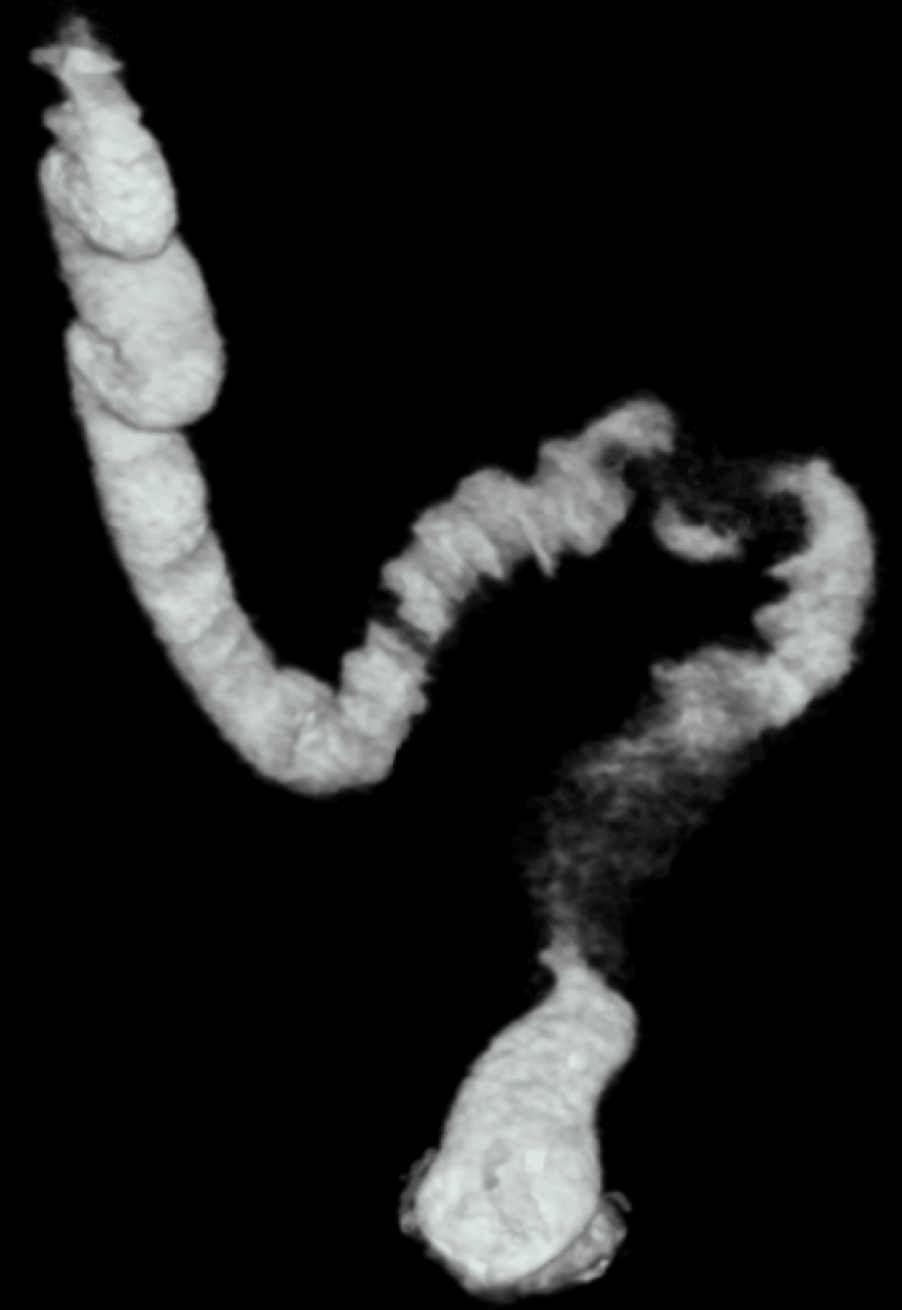
3D reconstruction of the contrast-enhanced abdominopelvic CT scan It shows dilation of proximal colonic loops and a constrictive ring in the sigmoid colon with fibrosed peritoneal tissue.

A prior pelvic ultrasound had documented a left ovarian endometrioma. In the preoperative setting, an abdominal contrast-enhanced CT scan was performed, which revealed dilated bowel loops with an abrupt change in caliber at the level of the sigmoid colon, and focal thickening of the wall was observed, forming a heterogeneous soft tissue mass, findings that may mimic colorectal cancer (Figure 1). A preoperative transvaginal ultrasound was not performed due to the presence of obstructive gastrointestinal symptoms.

Pelvic MRI (no citation) showed annular thickening of the affected segment, without mucosal invasion. These findings were consistent with infiltrating endometriosis (which typically invades the serosa and muscles, causing stricture).

**Figure 2:**
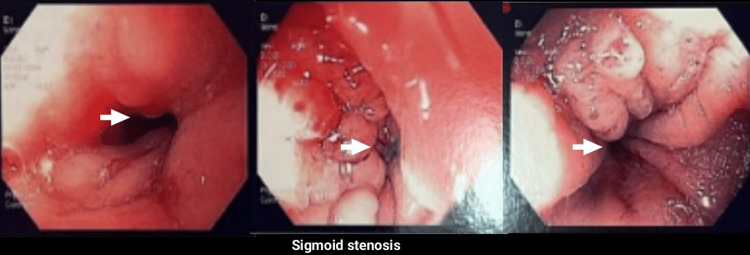
Colonoscopic findings of sigmoid stenosis Colonoscopy shows a segmental stenosis of the sigmoid colon with marked luminal narrowing due to extrinsic compression (arrows). The mucosa appears intact, with no ulceration or vegetative lesions, a finding compatible with intestinal endometriosis but not pathognomonic, as similar appearances may be observed in adhesions of other etiologies.

As shown in Figure 2, colonoscopy demonstrated a segmental sigmoid stricture with significant luminal narrowing caused by extrinsic compression, while the mucosa remained intact. This is consistent with reports in the literature, where colonoscopy is generally of low diagnostic yield in intestinal endometriosis, as the colonic epithelium is usually healthy. Mucosal biopsies were not taken due to the absence of ulceration and the low diagnostic yield of colonoscopic biopsies in this setting.

Histopathology

**Figure 3:**
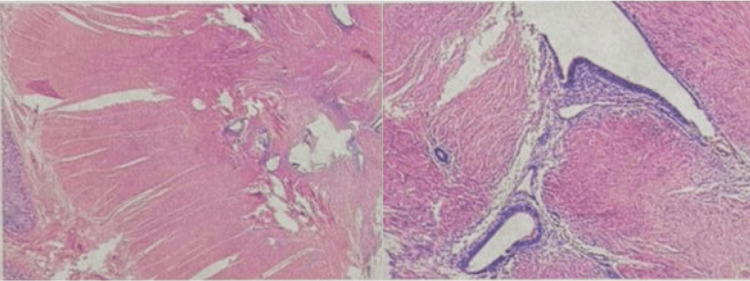
Histopathological findings of intestinal endometriosis Histopathological examination of the resected sigmoid colon showing ectopic endometrial glands and stroma infiltrating the muscularis propria, associated with marked fibrosis of the muscular layers (hematoxylin and eosin stain).

The pathological study of the intestinal resection revealed foci of ectopic endometrium (endometrial glands and stroma) infiltrating the muscularis of the intestinal wall with marked fibrosis of the muscular layers. In-depth examination, endometrioid-type glandular islands surrounded by endometrial stroma and fibrous reaction were identified, definitive findings of intestinal endometriosis (Figure 3). No malignant atypia were observed.

Problems in the diagnostic process

In 2015, the patient underwent exploratory surgery due to suspected acute appendicitis. During the procedure, endometriotic implants were identified; however, no tissue samples were sent for histopathological examination, precluding early diagnostic confirmation. One year later, pelvic ultrasonography revealed a left ovarian endometrioma, which was initially managed conservatively before subsequent surgical resection due to symptom progression.

Although endometriosis was already known and symptoms were cyclical, gastrointestinal manifestations, such as constipation alternating with diarrhea, were initially attributed to pelvic endometriosis and dysmenorrhea rather than intestinal involvement, delaying further investigation. In addition, deep infiltrating endometriosis may present without clear findings on noninvasive examinations; for example, routine colonoscopy often demonstrates preserved mucosa, as described in a series of patients with colonic endometriosis. The need for a high index of suspicion and limited access to advanced diagnostic techniques further contributed to the delayed recognition of bowel involvement.

Clinical reasoning

The presumptive diagnosis was established by integrating the gynecological history and the presenting findings. The patient had classic symptoms of deep endometriosis (severe dysmenorrhea, chronic pelvic pain, and constipation), along with a history of previous surgeries for pelvic endometriosis. Radiological images showed an intestinal stricture with an extraluminal mass (sudden changes in caliber and a hyperdense lesion on CT), with no intraluminal lesion detectable by colonoscopy, which almost certainly ruled out mucosal neoplasia. Given this evidence and the known pathophysiology of infiltrating endometriosis (which implants in the serosa and invades the muscularis, causing a stricturing mass), the probable diagnosis of intestinal stricture secondary to endometriosis was raised. Confirmation was only obtained with histology after surgical resection, as recommended in published guidelines and expert consensus, in which the definitive diagnosis is based on anatomopathological examination [5]. The diagnostic approach also considered differential diagnoses such as Crohn's disease and colorectal tumors, but the association with the underlying gynecological disease was decisive.

Therapeutic intervention

The patient underwent elective surgical management after conservative treatment options were considered but deemed unsuitable due to symptomatic intestinal stenosis secondary to deep infiltrating endometriosis. The presence of obstructive gastrointestinal symptoms and suspected fibrotic bowel involvement suggested a low likelihood of response to medical therapy alone. A multidisciplinary surgical approach involving general surgery and gynecology was planned. Intraoperatively, dense pelvic adhesions and a fibrotic stenotic segment of the sigmoid colon were identified. A segmental sigmoid colectomy with primary anastomosis was performed, achieving complete resection of the affected bowel segment. The postoperative course was uneventful, with progressive resolution of obstructive gastrointestinal symptoms. No immediate surgical complications were reported. Given the advanced stage of disease, postoperative gynecological follow-up was recommended to evaluate the need for adjuvant hormonal therapy aimed at reducing the risk of recurrence.

Follow-up and outcomes

During postoperative follow-up, the patient showed a favorable clinical evolution. Gastrointestinal symptoms, including constipation and abdominal distension, resolved completely after surgery. The patient reported significant improvement in pelvic pain and overall quality of life. No signs of anastomotic complications or recurrent obstruction were observed. Long-term follow-up was established with both gynecology and general surgery services to monitor disease recurrence and manage fertility-related concerns. Histopathological confirmation of intestinal endometriosis supported the appropriateness of the surgical approach and explained the preoperative clinical presentation.

Prognosis

The patient is considered to have grade IV deep endometriosis (disseminated with multiple pelvic implants, prior ovarian resection, and bowel obstruction), a condition characterized by extensive fibrosis and adhesions that may result in bowel involvement and obstruction [8]. The severity of endometriosis was classified according to the revised American Society for Reproductive Medicine (rASRM) classification (Table 2), which is freely available for clinical and academic use [9]. This advanced stage entails a complex surgical prognosis, as the surgery is often extensive and carries a reported recurrence risk of approximately 10-20%, particularly in cases of advanced disease [5]. In our case, the patient had previously achieved pregnancy after earlier gynecologic surgeries; however, her future fertility may be limited by the prior left oophorectomy performed during previous surgical management for ovarian endometriosis and by the severity of the disease. After conservative surgery for deep endometriosis, pregnancy rates are reported to be around 50-65%. Optimal management includes adjuvant medical treatment, such as postoperative hormonal suppression, to reduce the risk of recurrence and alleviate residual symptoms, along with long-term multidisciplinary follow-up. In general, bowel resection significantly improves quality of life by resolving the obstruction, but the long-term prognosis depends on the residual extent of the disease and the response to postoperative hormonal therapy. In this case, no hormonal treatment was administered in the preoperative period prior to intestinal resection.

**Table 2:** Revised American Society for Reproductive Medicine (rASRM) classification of endometriosis This table summarizes the revised American Society for Reproductive Medicine (rASRM) classification of endometriosis [9], stratifying disease severity into four stages based on lesion extent, depth, and adhesions.

Stage	Score (points)	Description
I (Minimal)	1–5	Superficial peritoneal implants, few and small lesions, minimal adhesions
II (Mild)	6–15	More and deeper implants, limited adhesions
III (Moderate)	16–40	Presence of endometriomas, more extensive implants, filmy or dense adhesions
IV (Severe)	>40	Large endometriomas, extensive deep implants, dense adhesions, obliteration of pelvic spaces

## Discussion

Intestinal endometriosis is an uncommon but clinically significant manifestation of deep infiltrating endometriosis, with a reported prevalence of 3-12% among women with endometriosis, increasing to up to 20% in advanced disease [2,7]. The rectosigmoid colon is the most frequently affected segment, accounting for approximately 60-90% of cases, which explains the predominance of lower gastrointestinal symptoms such as constipation, dyschezia, and cyclic abdominal pain [2,6].

Because mucosal involvement is rare, colonoscopic findings are often nonspecific and typically demonstrate extrinsic compression with preserved mucosa, as observed in this case [8]. This characteristic frequently delays diagnosis and may lead to misinterpretation as functional bowel disorders, inflammatory bowel disease, or colorectal malignancy. Imaging modalities, such as computed tomography and magnetic resonance imaging, play a crucial role in identifying bowel wall thickening, stenotic segments, and associated pelvic disease, although definitive diagnosis relies on histopathological confirmation following surgical resection [10,11].

Deep infiltrating endometriosis classified as revised ASRM (rASRM) stage IV is associated with extensive fibrosis, adhesions, and anatomical distortion, which may result in progressive luminal narrowing and, in rare cases, bowel obstruction [8]. Complete intestinal obstruction secondary to endometriosis is uncommon, occurring in less than 1% of cases, highlighting the clinical relevance of this presentation [9].

Surgical management is indicated in patients with significant stenosis, bowel obstruction, or refractory symptoms. Segmental bowel resection performed in specialized centers has demonstrated improvement in gastrointestinal symptoms and quality of life, although it carries a risk of postoperative complications and disease recurrence [5,11]. Consequently, long-term multidisciplinary follow-up and consideration of preoperative and/or postoperative hormonal therapy are essential to optimize outcomes and reduce recurrence rates [9].

## Conclusions

Intestinal endometriosis represents an uncommon but clinically significant manifestation of deep infiltrating endometriosis. Its infiltrative nature may lead to relevant stenosis of the gastrointestinal tract, with potential progression to partial or complete bowel obstruction. The case presented illustrates the diagnostic challenges inherent to this condition, which may delay timely diagnosis, particularly when endoscopic findings reveal an intact mucosa and the disease is confined to the deeper layers of the intestinal wall. The integration of clinical features, imaging studies, and histopathological confirmation allowed for the definitive diagnosis of colonic stenosis secondary to infiltrating endometriosis. This report highlights the importance of maintaining a high index of suspicion in women of reproductive age presenting with cyclical gastrointestinal symptoms and a history of pelvic endometriosis, as well as the need for a multidisciplinary approach to optimize diagnosis and management. Segmental intestinal resection, performed in specialized centers, with the aim of removing the stenotic segment responsible for symptoms, represents an effective therapeutic option for the relief of obstructive symptoms and improvement of quality of life; however, close follow-up remains essential due to the risk of recurrence.
